# Resting Energy Expenditure and Protein Balance in People with Epidermolysis Bullosa

**DOI:** 10.3390/nu11061257

**Published:** 2019-06-03

**Authors:** Ana Paula Zidorio, Camille Togo, Rosie Jones, Eliane Dutra, Kenia de Carvalho

**Affiliations:** 1 Graduate Program in Human Nutrition, Faculty of Health Science, University of Brasilia, Campus Universitário Darcy Ribeiro; Brasília 70910-900, Brazil; camillecristogo@gmail.com (C.T.); eliane.unb@gmail.com (E.D.); kenia@unb.br (K.d.C.); 2 Department of Dietetics, Birmingham Children’s Hospital, Birmingham B4 6NH, UK; rosiejones1@nhs.net

**Keywords:** epidermolysis bullosa, nutrition, energy expenditure, nitrogen balance, catabolism

## Abstract

Epidermolysis bullosa (EB) is a group of conditions characterized by severe fragility of the skin that causes recurring blistering. The recessive dystrophic subtype of EB (RDEB) has a strong impact on the nutritional status. We evaluated the resting energy expenditure (REE) and presence of protein catabolism in patients with RDEB. REE was assessed in 10 subjects (7 females; age range 4–33 years) by indirect calorimetry and using a predictive equation. Nitrogen balance was calculated by protein intake and 24 h urinary urea excretion estimations. An assessment of body surface area (BSA) with infected and non-infected skin lesions was applied to the nitrogen balance burn equation that was adapted to EB. The REE values predicted by the equation were consistently lower than the ones measured, except for two subjects. All subjects recorded high protein and energy intake, with protein intake being higher than 4 g protein/kg/day for five subjects. Even so, protein catabolism was observed in six subjects, three of whom had infected wounds. This study raises the hypothesis that the clinical and nutritional risks of people with RDEB are associated with an increased REE and negative nitrogen balance, which reinforces the importance of nutritional support.

## 1. Introduction

Epidermolysis bullosa (EB) is a rare group of diseases characterized by skin fragility due to mutations in several structural proteins, which causes the repeated development of blisters [1,2,3]. EB subtypes are classified into four major groups, including EB simplex (EBS), Junctional EB (JEB), Dystrophic EB (DEB) with dominant (DDEB) or recessive (RDEB) types, and Kindler syndrome (1).

RDEB is one of the most severe types of EB that affect the nutritional status [4,5]. People with RDEB have a limited ingestion and possibly absorption of nutrients [6]. Nutritional needs are increased when there is blistering because of the loss of body fluids rich in minerals and proteins as well as the requirements associated with wound healing and infection fighting. Additionally, any damage to the skin removes the external protective barrier, which permits the dissipation of heat and water. Subsequently, this increases the energy requirements for maintaining normal body temperature. The association of factors that limit the intake and absorption of nutrients with those that increase nutritional needs can lead to anaemia, decreased immunocompetence, poor wound healing, increased risk of infection, and malnutrition [4]. The clinical and metabolic conditions of EB have been compared to those post-burn because of the presence of open skin lesions, increased infection risk, heat loss, increased protein turnover, and high energy expenditure [6,7,8]. Gamelli [9] was the pioneer in associating the hemodynamic response observed post-burn with the conditions present in EB. In burn sufferers, the relationship of the proportion of burned surface area with resting energy expenditure (REE) and protein catabolism is well known [10,11,12,13]. In the case of people with EB, REE appears to increase proportionally to the body surface area with blister involvement [7], and the supply of dietary energy and protein must be high in order to reduce catabolism and promote growth [4,7,14,15].

Until now, there has been scarce information available about energy expenditure and protein catabolism in EB, with the majority based mainly on clinical practice and the use of predictive equations [4,14,16]. Only one case study performed indirect calorimetry in three children with JEB and RDEB [8], and, in another study [17], REE was measured by indirect calorimetry in an adult. No studies with nitrogen balance evaluation in people with EB were identified. The aim of this study was to investigate the measured and predictive REE and the presence of catabolism in subjects with RDEB. This study tested the hypothesis that people with RDEB experience protein catabolism. The predictive REE underestimated the measured REE and, therefore, it was not accurate in determining the nutritional requirements.

## 2. Materials and Methods

### 2.1. Type of Study and Subjects

A cross-sectional study focused on 10 subjects with RDEB who were attending the Nutrition Outpatient Clinic at the Brasilia University Hospital. The determination of the EB type was based upon clinical findings and electron microscopy of a skin biopsy sample. Subjects at least 4 years of age were included regardless of the extent of skin wounds. Patients under 4 years of age were excluded because of the constraints required during indirect calorimetry, and those with proteinuria were excluded because of possible errors in nitrogen balance results.

**Figure 1:**
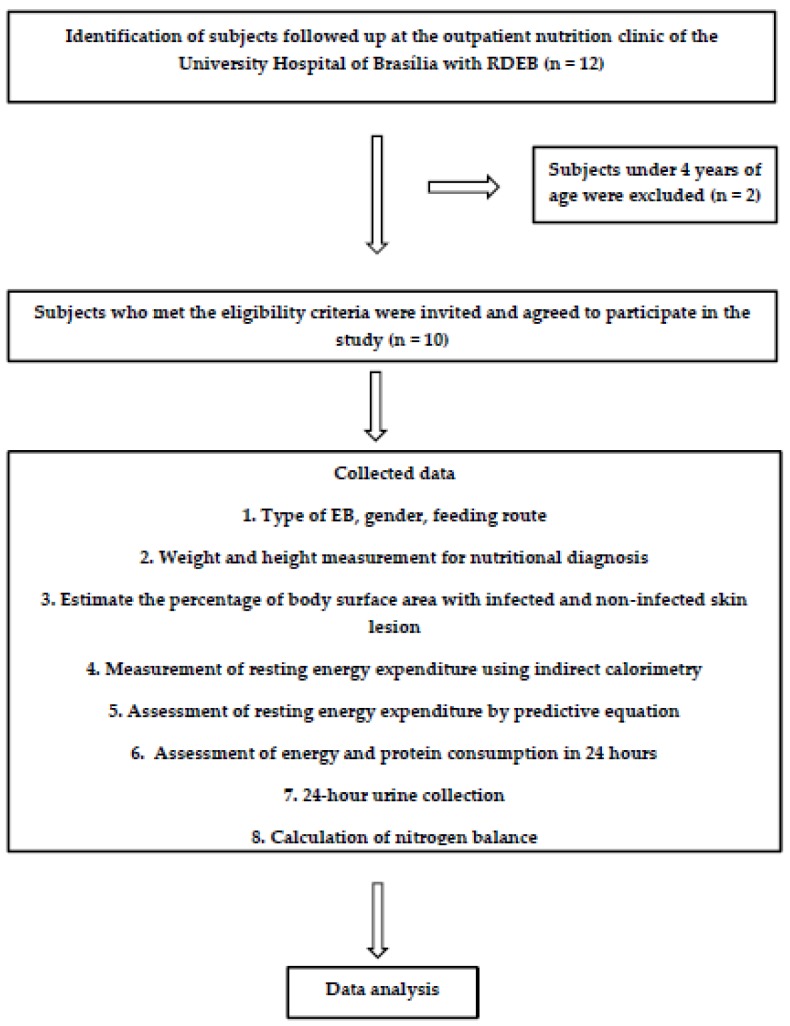
Study procedures. EB: epidermolysis bullosa, RDEB: recessive dystrophic EB.

The Human Research Ethics Committee of Health Sciences Faculty of the University of Brasília (protocol n. 1674735) approved this study. Signed informed consent and assent, when appropriate, were obtained from all subjects. Figure 1 describes the sample process and procedures involved in the study.

### 2.2. Nutritional Status

The nutritional status was evaluated on the basis of anthropometric data. Because of the fragility of the subjects’ skin, the anthropometry measurements included weight and height measurements without the use of instruments that compressed the skin. The subjects wore light clothing and were barefoot for the anthropometric evaluation. The height/age index (H/A) was evaluated using the percentile (*p*) proposed by the World Health Organization (WHO) [18] according to gender. The body mass index (BMI) was calculated to determine the nutritional status according to age [18,19,20].

### 2.3. Estimation of Percentage of Infected and Non-Infected Skin Lesions of Body Surface Area (BSA)

**Figure 2:**
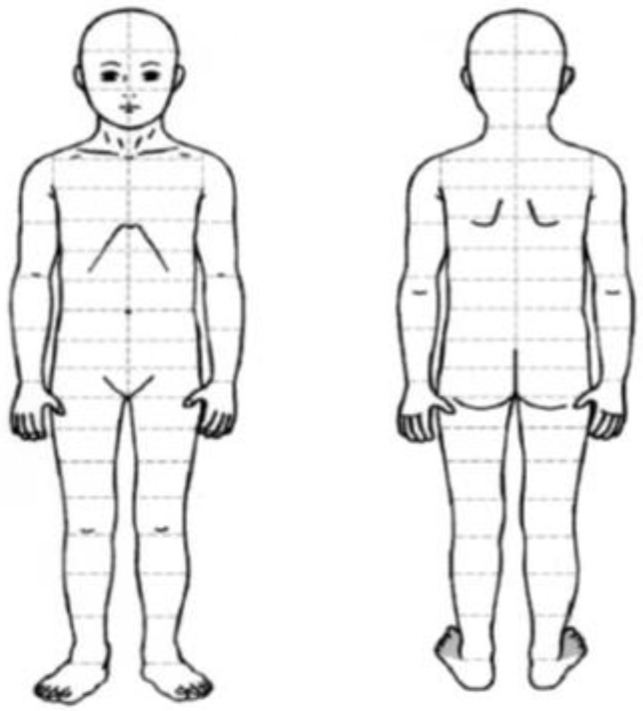
Form to calculate the percentage of body surface area (BSA) with skin lesions.

Estimates of the percentage of BSA (% BSA) lesions were based on the EB Clinical Practice Guidelines for Nutrition Support [14]. This form contains a representative image of a person from back and front that is subdivided into 100 small rectangles, each representing 1% of the body surface (Figure 2). The subjects or caregivers were instructed to color the areas either yellow or pink to represent those with infected or non-infected skin lesions, respectively. The calculation of % BSA wounded and infected was based on the sum of the areas shaded yellow or pink. Form filling was performed at the time of dressing change on the eve of the indirect calorimetry test.

### 2.4. Resting Energy Expenditure Measured by Indirect Calorimetry

The calorimeter uses the amount of inspired and expired gas exchanges to calculate energy expenditure [21]. The canopy system was used during the test with no compression mask in order to utilize a light comfortable device with no pressure or contact with the subject’s face. The participant was placed in the supine position, was free of physical and psychological stress, and was fasted and awake [22]. To guarantee the quality of measurement, the first 10 minutes (period for stabilization) of each indirect calorimetry test were discarded, and the mean of the last 20 min was used to calculate the REE [23]. The output included the estimation of 24 h REE and the corresponding respiratory quotient (RQ).

### 2.5. Resting Energy Expenditure Estimated by Predictive Equation

The Oxford equation [24] was used to define the predicted REE (Table 1). This equation is derived from the Schofield equation [25], which is adapted for tropical countries. For subjects under 17 years of age who presented a H/A below the 3rd centile, age correction was performed [16], using the age at which the current measured height corresponded to the 25th centile of the WHO growth curves [18].

### 2.6. Energy and Protein Intake

To assess dietary energy and protein intake, the subjects were instructed to complete a food record form. An explanatory note was provided in order to advise on how to describe consumed foods and methods of preparation and quantify ingested foods. The software *CalcNut* [26] containing the Brazilian Table of Food Composition [27] was utilized for intake calculations. The total energy and protein intake in grams (g) and per kilogram (kg) of body weight in 24 h was calculated.

### 2.7. Nitrogen Balance

For the calculation of nitrogen balance, the predicted equation for burns was adapted (9) to consider the percentage of BSA with second and third degree burns as the percentage of wounded/blistered BSA and infected BSA, respectively. The estimation of urinary urea and urinary urea nitrogen was determined by 24 h urine collection concomitant to food record completion. Urine analysis was performed by the Kjeldahl method [28]. To ensure the complete collection of urine and prevent forgetfulness, a specific container and reminder label (affixed to their underwear) were provided. The participants received verbal and written guidance regarding the correct collection and storage of urine. They were also advised to record the start and end times of the collection, whether oral or topical medication was used on the day of the collection (record of name and quantity), and whether there were any problems during the collection. For the collection, the first morning urine was discarded, and all other urine during the day, including the first morning urine of the following day, were collected. All collections were on Sundays because of the difficulty of collection on the day of class or work. The entire urine collection was checked by the research team.

The following equation was used to calculate nitrogen balance (NB):

NB (g) = (protein intake 24 h (g)/6.25) − {UUN (g/24 h) + 4 [(0.2 g × % BSA infected) + (0.1 g × % BSA wounded/blistered)]}

where UUN is urinary urea nitrogen.

The subjects who presented negative results of nitrogen balance were considered to be experiencing protein catabolism, and those with positive values were considered to be experiencing protein anabolism. The subjects whose results of the equation were between −1 and +1 were considered to be nitrogen equilibrium.

### 2.8. Data Analysis

The results were expressed in absolute values, per kg/body weight, or as a percentage for each participant. A plot of measured REE/predicted REE difference (Y-axis) for each equation against the mean of the measured REE and the predicted REE (X-axis) was created to assess the agreement between indirect calorimetry and prediction equation following the Bland–Altman method [29].

## 3. Results

Ten people (seven females) with RDEB were evaluated, with ages ranging from 4 to 33 years. The sociodemographic characteristics, growth data, and nutritional status are presented in Table 2. All subjects presented with malnutrition, which was characterized by a BMI below the recommended values. Three of the six subjects under 17 years had a H/A below the 1st centile, two were between the 1st and the 3rd percentile, and one between the 3 and the 5th percentiles. The difference between the chronological and corrected age ranged from 1 year and 4 months to 5 years and 6 months. All subjects were orally fed, and only one (subject #7) did not use a high energy/protein supplement.

**Figure 3:**
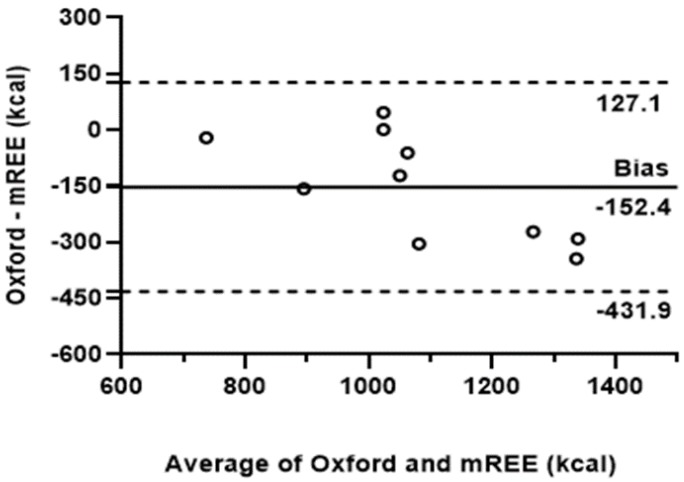
Agreement between the Oxford predictive equation and indirect calorimetry of subjects with RDEB (*n* = 10). m-REE: measured energy expenditure.

Table 3 shows the results of measured and predicted REE as well as nitrogen balance. The REE values predicted by the equation were consistently lower than the values measured, except for two adolescents (subjects #5 and #6). The agreement between the predicted equation and indirect calorimetry is detailed in Figure 3. There was poor agreement between the measured and the predicted REE. The Oxford equation underestimated the REE by a mean of 152.4 kcal (limits of agreement: 127.1 and −431.9 kcal).

Regarding the nitrogen balance results, it was not possible to evaluate subject #5 because of the subject’s known nephropathy. All subjects had a high protein intake, with six subjects having an intake higher than 4 g protein/kgW. Even with their high protein intake, six subjects, with the greatest % BSA lesions, were catabolic (negative nitrogen balance). All subjects with infected skin lesions were catabolic.

The energy intake ranged from 43 kcal/kgW to 135 kcal/kgW. All subjects consumed more energy than recommended for their age and gender population. The two subjects with the highest energy consumption per kilogram of weight were anabolic.

## 4. Discussion

This is the first study to evaluate both the measured REE and the nitrogen balance in a group of people with a severe type of EB. Serious nutritional compromise was observed as well as increased energy demand and protein catabolism, which were consistent with the severity of the disease in terms of BSA with skin lesions. These results reinforce the importance of clinical and nutritional management for these patients.

The analysis of the obtained REE data suggests that the Oxford equation is not adapted enough for predicting REE in people with EB, since only two cases had no underestimation of the predicted values. The Bland–Altman plot from the equation and calorimetry showed negative and highly heterogeneous values of measured REE and predicted REE, reflecting the fact that patients with RDEB have increased REE. Our results are consistent with those observed in studies with burns, which identified that the predictive equations underestimate the REE. In a study focusing on subjects with burns [23], the comparison of values measured by indirect calorimetry and predictive equations, as reported by Schofield [25], Harris and Benedict [30], and Food and Agriculture Organization/World Health Organization [31], revealed that in both sexes and in all age ranges, the values resulting from the three equations were lower than the measured ones. Focusing from this same perspective, the experts reported that patients with the most severe types of EB present an increased energy expenditure according to their clinical conditions, on the basis of their clinical practice [14,15,32,33]. Lechner-Gruskay et al. [7] evaluated the REE by indirect calorimetry in two children with RDEB and one with JEB. In all cases, the REE obtained by the predicted equation underestimated the measured REE, which agrees with the results for most subjects in the present study.

The predictive equations for REE may be accurate if the difference between the predicted and the measured results is less than 10% [34]. For adult subjects, all cases were above this limit. In a case report, Bonada et al. [17] observed that the REE estimated by the Harris–Benedict equation (30.2 kcal/kgW) underestimated the REE measured by calorimetry (57 kcal/kgW). In our subjects, the measured REE varied from approximately 30 to 50 kcal/kgW, and this variation may be due to differences in % BSA with lesions. For example, the subject with the highest REE measured by calorimetry (subject #9) was the one with the highest % BSA with infected lesions (16%). The report by Bonada et al. [17] contains no data about the size of the lesions. It is worth mentioning that the excessive energy expenditure in patients with EB reflects the inflammatory and catabolic state and greater heat production necessary to maintain body temperature because of the presence of open wounds [6].

In our study, the RQ ranged from 0.82 to 0.96. Some calorimeters only measure oxygen consumption and carbon dioxide production to calculate the energy expenditure, assuming that the RQ is a fixed value (0.8 or 0.85). In our study, the results of RQ validated the performed tests, since the quotients were between 0.67 and 1.3, which are within physiological values [21,35].

The nutritional recommendations proposed by the Institute of Medicine [31] established that the additional energy required to allow tissue deposition at a suitable rate for health is 20–25 kcal/day for boys and girls from 3 to 18 years of age. The majority of our subjects had a high energy intake. However, only children who consumed around 130 kcal/kgW were in an anabolic state. Considering that people with RDEB are underweight and require a greater supply of energy [4,14], a deficit of more than 15% of predicted REE, such as that observed in 50% of this sample, may lead to an underestimation of the nutritional needs, especially for children under stress conditions [36].

Nitrogen balance is an accurate indicator of metabolic stress [37], and it is expected that in healthy people with adequate protein consumption, nitrogen balance will have a zero value [38]. The technique requires the quantification of all nitrogen intake and loss routes [38]. In the case of EB, an estimation of protein loss through the skin is essential for more accurately determining the balance value [9]. In a pioneering study about the metabolic profile in people with EB, Lechner-Gruskay et al. [7] suggested that, despite the lack of information about the measurement of cutaneous protein loss, it is still important to consider this in future studies, since it is quite difficult to maintain a positive balance. In our study, we found that six of the nine subjects evaluated were catabolic. Consequently, it is presumable that the protein requirements of people with EB are significantly higher than those of the healthy population because of the substantial loss of protein through blisters and increased protein synthesis for tissue repair and inflammatory processes [15]. Those subjects with a protein intake of 4–5 g/kgW presented lower protein catabolism or were in equilibrium. This may indicate that even with the presence of large BSA with skin lesions, it is possible to attenuate protein catabolism with a high protein intake. The experts suggest that the protein intake should be up to 200% of the recommended intake in order to improve the nutritional status of children with EB [14,39].

Some limitations of this research need to be identified. First, the small number of subjects and the limited age range due to the rarity of the disease did not allow a robust statistical approach. Second, an analysis of the usual food intake was not performed, which made it difficult to control for possible confounding factors. It was not possible to assess the adequacy of energy consumption because of the lack of specific reference values. However, it was identified that the subjects consumed more energy than recommended for the general population. Finally, the cross-sectional design of the study prevented the evaluation of causality between the variables. Further studies are needed to evaluate the impact of % BSA with skin lesions, infected or not, on the metabolism of children and adults with EB. Furthermore, there should be intervention studies performed to investigate the effect of diet on the clinical manifestations of the disease.

It was possible to observe that REE was generally underestimated by the predictive equation, and although the subjects had a high protein intake, most of them were catabolic, which was consistent with the presented size of BSA with lesions. Nitrogen balance assessment can be performed in most healthcare settings around the world. In services where a calorimeter is not available, the recommendation of 100–150% [4,14] of energy requirements is a safe alternative for people at nutritional risk and with the most severe types of the disease. Given the scarcity of studies of this serious disease, our results highlight the importance of routinely evaluating the energy and protein metabolism of patients with RDEB to ensure a better nutritional status.

## Figures and Tables

**Table 1:** Resting energy expenditure (REE) predicted by the Oxford equation.

Age	Female (Kcal/day)	Male (Kcal/day)
3–10 years	20.1 × Weight + 507	23.3 × Weight +514
10–18 years	11.1 × Weight + 761	18.4 × Weight + 581
18–30 years	13.1 × Weight + 558	16.0 × Weight + 545
30–59 years	9.74 × Weight + 694	14.2 × Weight + 593

**Table 2:** Sociodemographic characteristics, growth, and nutritional status of subjects with recessive dystrophic epidermolysis bullosa.

Patient	Gender	Age	Corrected Age *	H/A (Percentile)	BMI (kg/m^2^)	Nutritional Status **
1	F	4 y 8 m	3 y 3 m	1–3°	11.9	severe thinness
2	F	7 y 7 m	6 y 3 m	1–3°	12.0	thinness
3	M	12 y 11 m	7 y 11 m	<1°	13.4	thinness
4	F	13 y	9 y 9 m	<1°	14.5	thinness
5	F	16 y 7 m	11 y 1 m	<1°	10.3	severe thinness
6	F	16 y 7 m	12 y 10 m	3–5°	12.9	severe thinness
7	M	21 y	NA	NA	17.6	thinness
8	F	22 y	NA	NA	16.7	severe thinness
9	M	23 y	NA	NA	12.2	severe thinness
10	F	33 y	NA	NA	17.5	thinness

BMI: body mass index; M: male; F: female; y: years, m: months; NA: not applicable; * age correction considering age at the current height at p25 of the WHO growth curves; ** thinness: ≤17 years with BMI: 1–3° and adult with BMI: 17–18.4 kg/m^2^; severe thinness: ≤17 years with BMI <1° and adult with BMI <17 kg/m^2^.

**Table 3:** Measured and predicted resting energy expenditure, energy intake, nitrogen balance, and their components in subjects with RDEB.

Patient	Gender	Corrected Age *	% BSA with Non-Infected Lesions	% BSA with Infected Lesions	Respiratory Quotient	REE Predicted **		REE Measured by IC		Energy Intake (kcal/kg)	Protein Intake		Urinary Urea Nitrogen (g/day)	Nitrogen Balance (g)	Nitrogen Balance Conclusion
						kcal/day	kcal/kg	kcal/day	kcal/kg		g	g/kg Body Weight			
1	F	3 y 3 m	15	0	0.89	726	67	747	68	75	44	4.01	2.91	−1.91	catabolic
2	F	6 y 3 m	13	3	0.82	816	53	973	63	95	81	5.24	2.78	−2.52	catabolic
3	M	7 y 11 m	20	0	0.89	989	48	1111	54	130	85	4.18	3.42	2.23	anabolic
4	F	9 y 9 m	22	0	0.90	1032	39	1093	42	135	138	5.27	5.17	8.05	anabolic
5 ^a^	F	11 y 1 m	20	0	0.82	1047	41	1000	39	130	149	5.79	NA	NA	NA
6	F	12 y 10 m	27	0	0.82	1024	43	1023	43	57	29	1.22	3.27	−9.42	catabolic
7	M	21 y	17	0	0.96	1164	30	1508	39	45	59	1.49	1.83	0.62	balance
8	F	22 y	28	4	0.93	1193	25	1484	31	59	109	2.25	8.63	−5.57	catabolic
9 ^b^	M	23 y	0	16	0.89	929	39	1233	51	74	99	4.12	4.71	−1.70	catabolic
10	F	33 y	23	7	0.83	1130	25	1402	31	43	70	1.56	4.68	−8.27	catabolic

REE: resting energy expenditure; y: years; m: months; kcal: kilocalories, kg: kilo; IC: indirect calorimetry; % BSA: body surface area; NA: not applicable; ^a^ patient with nephropathy; ^b^ wheelchair user; * age correction considering age at the current height at p25 of the WHO growth curves; ** Oxford equation for males 10–18 years: 18.4 W + 581; 18–30 years: 16.0 W + 54; females 3–10 years: 20.1 W + 507; 10–18 years: 11.1 W + 761; 18–30 years: 13.1 W + 558; 30–59 years: 9.74 W + 694; W = weight.
